# Public awareness of common eye diseases in Jordan

**DOI:** 10.1186/s12886-017-0575-3

**Published:** 2017-10-02

**Authors:** Mera F. Haddad, May M. Bakkar, Nour Abdo

**Affiliations:** 1 Faculty of Applied Medical Sciences, Department of Allied Medical Sciences, Jordan University of Science and Technology, PO Box 3030, Irbid, 22110 Jordan; 2 Faculty of Medicine, Department of Public Health, Jordan University of Science and Technology, Irbid, Jordan

**Keywords:** Awareness, Eye, Disease, Jordan

## Abstract

**Background:**

Cataract, glaucoma, diabetic retinopathy and dry eye disease are common with high prevalence in Jordan. This study aims to assess the awareness of these ocular diseases among Jordanian population.

**Method:**

A self-designed questionnaire was developed in Arabic and used to interview people in different provinces of Jordan. Socio-demographic data e.g. age, gender and level of education was reported. Public awareness of four ocular diseases; cataract, glaucoma, diabetic retinopathy (DR) and dry eye disease (DED) was assessed. Questions about familiarity with the diseases, familiarity with their risk factors and participants’ source of knowledge were asked. Moreover, awareness of the effect of these disease on the eye whether they are blinding, preventable, treatable and/or the vision is back to normal following treatment was also assessed.

**Results:**

A total of 802 participants (232 males and 570 females) completed the questionnaire. The average age (± standard deviation) of the study participants was 28 ± 11.6 (range 18 to 80 years old). Awareness of cataract, glaucoma, DR and DED was reported by 31%, 38%, 37% and 52% of the study population, respectively. Family/relatives/friends and mass media appeared to be the most common sources of knowledge. Age and level of education of the participants were significantly associated with the level of disease awareness.

**Conclusion:**

This work shows that the level of awareness of the four ocular diseases among Jordanians is good and compares with many reports in the developed and developing countries. Familiarity and knowledge about ocular diseases is essential as it would increase the chance of the subject being tested and thus diagnosed early enough if any problem occurred. Better understanding of the disease would encourage subjects to seek medical care sooner which in turn would prevent visual impairment. Therefore, awareness campaigns should be made to target unaware population.

**Electronic supplementary material:**

The online version of this article (10.1186/s12886-017-0575-3) contains supplementary material, which is available to authorized users.

## Background

Many ocular diseases such as glaucoma, cataract and diabetic retinopathy are considered leading causes of blindness worldwide. Poor health awareness of these conditions and their complications causes a delay in seeking medical care and chances of early intervention and prevention. Therefore, raising public awareness of ocular diseases plays a significant role in the early diagnosis and treatment of such conditions and thus reduces the burden of visual impairment [1, 2].

Variable results have been reported about the level of awareness of common ocular diseases worldwide [3–6]. In developed countries such as Canada, the level of awareness of ocular diseases was reported at 69% for cataract and 41% for glaucoma [5]. While in urban population in India knowledge about ocular diseases was poor. For instance in a report from southern India, the majority of patients (90%) with glaucoma were not aware of the condition and its complication [7, 8]. In another study in India a very poor awareness of glaucoma (3.2%) and diabetic retinopathy (27%) of the study population was also reported [1].

Studies have suggested many factors which may influence the level of awareness and knowledge about ocular diseases. Those include age, gender, education level, and socioeconomic status of people. For example, older people and females were suggested to have better knowledge of glaucoma and diabetic retinopathy [4]. These factors in addition to religion and education were found to influence peoples’ knowledge about glaucoma, cataract and diabetic retinopathy in India [1, 6]. The lack of education and poor socio-economic status not only were associated with poor knowledge of ocular diseases but also affected the awareness of vision loss prevention [9].

Awareness of ocular diseases should not only aim for better understanding of the disease but also encourage people to better utilize available eye care services. Utilization of health services in the country plays a significant role in the prevention of blindness due to ocular diseases. For example in Tehran, it was found that only 22% of diabetic patients had a history of regular eye examination [10] and large proportion of the population had never utilized any eye care services [11]. These results are not encouraging since the prevention and treatment would not be feasible without regular eye examination and early detection of the disease.

Awareness of ocular diseases has been reported in developed and developing countries. There is a lack of studies that assessed the awareness of ocular diseases and presbyopia in the Middle East region. Few reports have been published describing the awareness level of cataract, glaucoma and diabetic retinopathy for Iranian population. Up to the authors’ knowledge, there are no such reports that highlights this issue for Jordanian population. This study sought to assess public awareness of common ocular diseases such as glaucoma cataract, diabetic retinopathy and dry eye in Jordan.

## Methods

### Study design and participants

A semi-structured questionnaire (Additional file 1) was developed and modified by researchers based on previously published literature [1, 12]. The questionnaire was originally developed in English language and then translated to Arabic language using forward and backward translations and was administered to willing participants in Arabic form. Face and content validity was assessed by different faculty members in the Optometry and Ophthalmology Department at Jordan University of Science and Technology. The questionnaire was further reviewed by expert panel and then distributed for testing by a pilot group (*n* = 30) to further assess clarity and relevance of survey questions. Comments and feedback by the pilot group was taken into consideration before the final survey is distributed to target population. Data from pilot group was not included in the final analysis.

The study participants were selected randomly from the general population in different provinces in Jordan. Participants aged ≥18 years old and were approached to participate and informed consent was obtained from all participants prior to their participation in the study. A brief medical history was recorded to exclude participants with a history of any systemic diseases such as diabetes mellitus, hypertension or heart diseases. In addition, an ocular history was recorded to ensure that none of the participants had any of the of the four study diseases (cataract, glaucoma, diabetic retinopathy and dry eye disease) as this may influence the results and increase the level of awareness about that particular disease.

### Data collection

The questionnaire was administered to collect data on socio-demographics such as age, gender, and level of education. The participants were then asked to complete the questionnaire which assessed their knowledge about four ocular diseases: glaucoma, cataract, diabetic retinopathy (DR) and dry eye disease (DED). Awareness of these diseases was documented if the participant was able to describe the disease and mention one or more of its related symptoms. Participants were then required to report any risk factor for each of the four diseases. Even participants who were not able to describe the disease in the previous question, but had heard about it were required to answer this question. The following question required each participant to report the source of knowledge about the disease, whether it was from the ophthalmologist, optometrist, family and friends, media, books or the web. More questions followed about each disease in terms of being a blinding, preventable, treatable and whether the vision is restored after treatment.

### Data analysis

All data analyses were performed using SAS 9.2 (SAS Institute Inc., Cary, NC, USA). Descriptive frequencies and percentages were used to summarize categorical data. Univariate comparisons between awareness of each ocular disease and age were done using independent t-test and using. Chi-square tests for the categorical variables (gender and level of education). Fisher’s exact test was used for expected counts that were less than 5. Univariate analysis was followed by multiple logistic regressions where the effect of multicategorical variable was assessed by keeping the first category as the reference. The level of significance was set at (*p* < 0.05).

## Results

### Socio-demographics of participants

A total of 802 participants (232 males and 570 females) completed the questionnaire. The average age (± standard deviation) of the study participants was 28 ± 11.6 and it ranged from 18 to 80 years old. The level of education varied among subjects such that 17 (2.1%) participants were ‘illiterate’, 54 (6.7%) participants finished ‘elementary school’, 291 (36.3%) completed ‘high school’, 414 (51.6%) were ‘university graduates or undergraduates’ and 26 (3.2%) had higher education degree (masters or PhD). Participants were distributed in the northern, middle and southern of Jordan.

### Awareness of ocular diseases

**Fig. 1:**
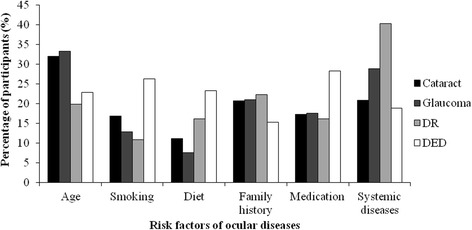
Participants’ awareness of risk factors related to the four diseases

**Fig. 2:**
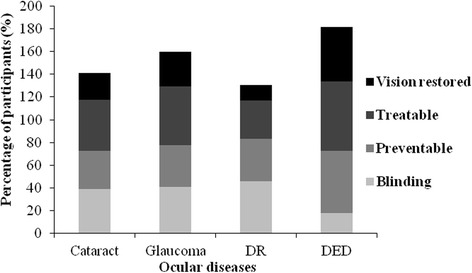
Participants’ awareness of the diseases’ (cataract, glaucoma, DR and DED) effect on the eye

Awareness of cataract was reported by 31.4% of the participants. Table 1 represents the number (%) of participants who were aware of the disease, its risk factors and its effect on the eye. The results show that age was the most common risk factor (32.0%) of the responses) reported for the disease followed by systemic disease and family history (~20% of the responses). The least common reported risk factor related to cataract was diet (11.1% of the responses). Figure 1 shows the awareness of risk factors related to the four diseases. Most of the participants (38.8%) reported that cataract is a blinding condition (Fig. 2). However, many of them (33.7%) reported that it could be prevented and 45.0% said that it could be treated.

**Table 1:** Responses of participants to questions related to the four ocular diseases (Cataract, Glaucoma, DR and DED) (*n* = 802)

	*Cataract*	*Glaucoma*	*DR*	*DED*
Question	No. (%)^a^	No. (%)^a^	No. (%)^a^	No. (%)^a^
Are you familiar with any of these eye diseases?	252 (31.4)	311 (38.8)	299(37.3)	416 (51.9)
Can you report any risk factor for the disease?				
Age	257 (32.0)	266 (33.2)	159 (19.8)	184 (22.9)
Smoking	135 (16.8)	103 (12.8)	87 (10.9)	211 (26.3)
Diet	89 (11.1)	61 (07.6)	129 (16.1)	186 (23.2)
Family history	166 (20.7)	168 (21.0)	178 (22.2)	123 (15.3)
Medication	139 (17.3)	141 (17.6)	129 (16.1)	226 (28.2)
Systemic diseases	167 (20.8)	231 (28.8)	323 (40.3)	151 (18.8)
About the condition^b^				
Is it blinding	311 (38.8)	325 (40.5)	369 (46.0)	141 (17.6)
Is it preventable	270 (33.7)	295 (36.8)	300 (37.4)	442 (55.1)
Is it treatable	361 (45.0)	419 (52.2)	268 (33.4)	490 (61.1)
Is the vision back to normal after treatment	191 (23.8)	244 (30.4)	111 (13.8)	293 (47.7)
Source of knowledge for the four diseases				
Family, friends and relatives	422 (52.6)			
Media e.g. TV, radio	284 (35.4)			
Internet	253 (31.6)			
books, newspaper, magazines	242 (30.2)			
Ophthalmology clinic	207 (25.8)			
Previous history	156 (19.5)			
Optometry clinic	136 (17.0)			

*DR* diabetic retinopathy, *DED* dry eye disease

^a^Number of participants who answered ‘yes’ for being aware of the disease

^b^Number of participants who thought that the disease is blinding, preventable, treatable and the vision is restored after treatment

Awareness of glaucoma was reported by 38.8% of the participants (Table 1). The most reported risk factors for glaucoma were similar in order to those reported for cataract with differences in the response rate. The age of the patient appeared to be the most common risk factor reported for the disease (33.2% of the responses) followed by systemic disease (28.8%) and family history (21.0%) (Fig. 1). Figure 2 shows that many of the participants (40.5%) reported that glaucoma is a blinding condition. However, almost half of them (52.2%) reported that it could be treated.

Awareness of diabetic retinopathy was reported by 37.3% of the participants (Table 1). The most reported risk factor in the case of DR was systemic disease (40.3%). followed by family history (22.2%) and age (19.8%) (Fig. 1). Nearly half of the participants (46.0%) reported that DR is a blinding condition and a reasonable number (37.4%) knew that it could be prevented and treated (33.4%). A small percentage (13.8%) reported that the vision goes back to normal after treatment.

Awareness of dry eye disease was reported by more than half of the study population (Table 1). The most common risk factor related to the disease was medication (28.2%) followed by smoking (26.3%) and diet (23.2%) (Fig. 1). When subjects were asked about the condition, only a small percentage reported that DED is a blinding condition. Most of the subjects (55.1%) thought that DED could be prevented and the majority (61.10%) reported that it could be treated (Fig. 2).

When subjects were asked about the source of their knowledge about the four diseases, the majority of them (52.6%) reported family, relatives and friends as the most common source of knowledge (Table 1). This was followed by media (35.4% of the responses) and internet (31.6%). The least common reported source of knowledge was the optometrist (17.0%).

### Association between the level of awareness of ocular diseases and study variables

Table 2 shows demographic association between the awareness of the four diseases with age, gender, and level of education. Student *t-test* was used for analyzing the association of awareness and the age of participants as continuous variable. There was a significant difference in the level of awareness between age groups for cataract (*p* = 0.02), DR (*p* = 0.01) and DED (*p* = 0.01) but not for glaucoma (*p* = 0.10). Multiple logistic regression analysis showed that awareness of cataract, glaucoma, and DR increased mildly but not significantly with increasing age. However, the opposite occurred for DED where participants of younger age knew more about the disease than older population (Table 3).

**Table 2:** Association of awareness of eye diseases with subject demography (age, gender and education) (n = 802)

	*Cataract*		*Glaucoma*		*DR*		*DED*	
	No. (%)^a^	*P-value* ^b^	No. (%)^a^	*P-value* ^b^	No. (%)^a^	*P-value* ^b^	No. (%)^a^	*P-value* ^b^
Age groups^c^ (years)								
<20	15 (31.9)	0.02	15 (31.9)	0.10	12 (25.5)	0.01	27 (57.5)	0.01
≥ 20–30	153 (28.9)		197 (37.2)		188 (35.5)		287 (54.3)	
≥ 30–40	28 (31.8)		38 (43.1)		33 (37.5)		45 (51.1)	
≥40–50	30 (35.7)		38 (45.2)		37 (44.1)		34 (40.5)	
≥ 50–60	17 (50.0)		16 (47.1)		21 (60.8)		16 (47.1)	
60 ≥	9 (45.0)		7 (35.0)		8 (40.0)		7 (35.0)	
Gender								
Males	66 (28.5)	0.28	90 (38.8)	1.00	89 (38.4)	0.69	113 (48.7)	0.28
Females	186 (32.6)		221 (38.8)		210 (36.8)		303 (53.2)	
Level of education								
None	10 (58.8)	0.02	8 (47.1)	0.0001	5 (29.4)	0.45	6 (35.3)	0.05
Elementary	17 (31.5)		28 (51.9)		24 (44.4)		21 (39.0)	
High school	76 (26.1)		149 (51.2)		107 (36.8)		158 (54.3)	
University	139 (33.6)		116 (28.0)		150 (36.2)		213 (51.5)	
Higher education	10 (38.5)		10 (38.5)		13 (50.0)		18 (69.2)	

^a^Number/% of participants who answered ‘yes’ for being aware of the disease

^b^Significance of the association between subject demographics and awareness of the four diseases

^c^Age was analyzed as a continuous variable

**Table 3:** Multiple logistic regression for the association of awareness of eye diseases with subject demography (age, gender and education) (n = 802)

	*Cataract*	*Glaucoma*	*DR*	*DE*
	AOR (95%CI)	AOR (95%CI)	AOR (95%CI)	AOR (95%CI)
Age^a^	1.02 (1.01,1.03)	1.01 (1.0,1.02)	1.02 (1.01,1.03)	0.98 (0.97,1.0)
Gender				
Male	Ref (1.00)	Ref (1.00)	Ref (1.00)	Ref (1.00)
Female	1.47 (1.01,2.13)	1.30 (0.90,1.83)	1.14 (0.80,1.61)	1.04 (0.74,1.45)
Level of education				
None	Ref (1.00)	Ref (1.00)	Ref (1.00)	Ref (1.00)
Elementary	0.35 (0.11,1.12)	1.31 (0.44,3.92)	2.12 (0.64,7.04)	1.15 (0.36,3.64)
High school	0.30 (0.10,0.80)	1.22 (0.45,3.13)	1.75 (0.58,5.28)	1.83 (0.65,5.19)
University	0.41 (0.15,1.15)	0.45 (0.17,1.22)	1.76 (0.58,5.30)	1.57 (0.56,4.44)
Higher education	0.47 (0.13,1.67)	0.73 (0.21,2.53)	2.66 (0.71,9.90)	4.03 (1.09,14.93)

*AOR* adjusted odds ratio, *CI* confidence interval, *Ref* reference

The first category was taken as a reference

^a^Age was analyzed as a continuous variable

Overall, females appeared to be more aware of ocular diseases (cataract, glaucoma, DR, and DED), although the difference in awareness between males and females was not significant neither with univariate analysis nor with multiple logistic regression (*p* > 0.2 for cataract, glaucoma DR and DED).

The results showed a significant association between the education and awareness of cataract (*p* = 0.02), glaucoma (*p* = 0.001) and DED (*p* = 0.05). However, familiarity with the diseases was not restricted for people with higher education. After adjusting for age and gender, lower education was associated with more awareness of cataract and glaucoma although it did not reach statistical significance. On other hand, increased education was significantly associated with more awareness of DED (Table 3).

## Discussion

Some studies have reported data about the awareness of different ocular diseases among the general population. Data from around the world has shown variations in the level of awareness about common ocular diseases. Unfortunately data in the Middle regarding this issue is lacking. This study looked to assess the awareness of four ocular disease; cataract, glaucoma, diabetic retinopathy and dry eye disease among Jordanian population.

Familiarity with the diseases in Jordan was mostly reported for dry eye disease. Nearly half of the study population reported knowledge of dry eye and described it very well. This could be attributed to the high prevalence of dry eye in Jordan according to our recent report [13] which showed that 59% of the selected population suffered from intense dry eye symptoms. Therefore the higher level of awareness could be due to people being affected with DED disease or experienced one or more of dryness symptoms at least once in their lives.

The level of awareness of glaucoma in this work was relatively high compared to other developing countries such as India. In southern India, the awareness of glaucoma was very poor and the majority of patients with glaucoma were not aware of the condition and its complication [7, 8]. A similar study for the same population also reported a very poor awareness of glaucoma (3.2%) compared to other diseases such as cataract [1]. Poor awareness of glaucoma was also reported for Chinese population where only 10% knew about the disease compared to 90% who could describe cataract for instance [14]. The results in this work agree more with studies reported in the developed countries like Australia [3] and Canada [5] where high level of awareness of glaucoma was reported (41.2% of the study population) [5]. Similar results were reported for Iranian population where 46.6% were aware of glaucoma and even more aware of cataract and DR (~80%) [12].

The high level of awareness among Jordanian people is probably due to the simple definition of glaucoma “increased pressure in the eye” rather than describing a “lens opacity” in the case of cataract. For cataract most people described cataract as a white membrane covering the eye, and thus this was recorded as unaware. Dandona et al. [1] reported similar lack of knowledge about cataract even though familiarity with the condition was high. This may also explain the higher level of awareness of diabetic retinopathy (37%) compared to 27% in south India [1] This is because the terminology in Arabic exactly describes the disease. Therefore, most people reported it as “retinopathy due to diabetes”.

The level of awareness of risk factors for all the diseases was acceptable. Older age, systemic diseases and family history were reported the highest as disease risk factors for glaucoma, cataract and diabetic retinopathy. Similar knowledge of risk factors was highlighted in a study on Chinese population [15] where older age and family history were the most common risk factors reported for glaucoma. Once again, in this work, the terminology could have played a role in deciding the risk factor since the definition of glaucoma and diabetic retinopathy can be related to systemic blood pressure and diabetes mellitus, respectively.

Many people knew that cataract, glaucoma and DR are blinding conditions. However, a reasonable percentage knew that these diseases can be treated. DR was considered the most blinding condition among the four diseases and the chance of restoring vision after treatment is low. This could be explained by the high prevalence of DR (64.1%) among diabetics in Jordan [16] and thus high awareness of its complications compared to other diseases like glaucoma and cataract. Awareness of treatment and prevention was more apparent for dry eye disease. Once again the prevalence of DED in Jordan and the possibility of experiencing dry eye in various situations and places may explain the high awareness of the condition.

This work has shown that the most common source of knowledge of all diseases was family, relatives and friends followed by mass media. The latter shows the efficiency of these sources for educating people. TV and radio have been shown to be the most effective methods of education and raising awareness. Baker and Murdoch [17] showed that the level of awareness among Indian population was increased from 22% to 69% after radio enlightment. Our results agree with reports from Germany [18] and southern India [1] where the most common sources of knowledge about glaucoma in particular was friends and media. A surprising finding in this work was that ophthalmologists and optometrists were the least reported source of knowledge about the diseases. This is very disappointing since these sources are the first body to communicate with the patients diagnosed with diseases such as glaucoma, DR and cataract. Isawumi et al. [19] suggested that the ophthalmologist should educate the patient about ocular diseases and they should encourage the patient’s companion to test their eyes in general and IOP in particular. In addition, education at the clinic should always be given in the waiting room prior to examining the eye.

The awareness of ocular diseases was significantly associated with the age and level of education of participants. Older people were more aware of cataract, glaucoma and diabetic retinopathy. However, in the case of dry eye younger people were significantly more aware of the condition. The latter could be explained by the life style adapted by young population recently, with the introduction of digital devices e.g. tablets and smart phones and the consistent use of computers, young people experience dry eye symptoms more than older people [20]. The use of these devices has become a serious phenomenon in Jordan among young population and children and it is alarming due to their anticipated effect on the eye.

The level of education was associated with awareness of ocular diseases except for diabetic retinopathy. Surprisingly, our results showed that people with decreased level of education < high school were more aware of cataract and glaucoma. This finding contradicts with most studies of ocular disease awareness in which higher level of education was correlated with higher awareness of the disease [2, 4, 9, 21]. Our results could be explained by the increased prevalence of diseases such as glaucoma, cataract and dry eye among people with lower socio-economic status. This plays a significant role in the awareness level i.e. the subject will become more aware of the disease when he/she has it, or when family members, relatives or friends in their neighborhood are affected. Increased education was associated with increased awareness of DR and DED. These contradicting results show that education is not a key determinant in the assessment of ocular diseases.

Interestingly, the overall awareness of ocular diseases was higher among females compared to males, although the difference was not significant. This could be attributed to the culture of the country where older females are usually not working and they accompany family members to hospitals when they seek medical help. This may contribute to their knowledge about diseases in general as they become fully aware of the case and able to care for their family members.

Although Jordan is considered a developing country, the level of awareness of ocular diseases was not bad considering the limited resources of the country. Jordan has crossed a milestone in the education and health domains. A very high percentage of the population has health insurance and efforts have been made to increase the level of children’s access to schools. This is beneficial in terms of increasing the general awareness of the population.

## Conclusion

In this study, a sample of Jordanians was recruited to assess their awareness level of common ocular diseases. Over all, the level of awareness of the four diseases was high. Participant’s age and level of education were the main factors found to be significantly associated with awareness of ocular diseases.

To the authors’ knowledge, this is the first study that assesses the awareness of common ocular diseases in Jordan. The data set from this study is of paramount importance because previous knowledge of the disease may encourage the patient to pursue medical care sooner and this in turn would reduce the burden of visual impairment in the society.

There is an imperative need to implement strategies to increase further awareness of ocular diseases to reduce the risk of visual complications.

## Additional file

Additional file 1: Questionnaire. (DOCX 25 kb)

## Abbreviations

AOR: Adjusted odds ratio
CI: Confidence interval
DED: Dry eye disease
DR: Diabetic retinopathy
Ref: Reference

## References

[1] Dandona R (2001). Awareness of eye diseases in an urban population in southern India. Bull World Health Organ.

[2] Shrestha MK (2014). Health literacy of common ocular diseases in Nepal. BMC Ophthalmol.

[3] Attebo K (1997). Knowledge and beliefs about common eye diseases. Aust N Z J Ophthalmol.

[4] Chew YK, Reddy SC, Karina R (2004). Awareness and knowledge of common eye diseases among the academic staff (non-medical faculties) of University of Malaya. Med J Malaysia.

[5] Noertjojo K (2006). Awareness of eye diseases and risk factors: identifying needs for health education and promotion in Canada. Can J Ophthalmol.

[6] Sathyamangalam RV (2009). Determinants of glaucoma awareness and knowledge in urban Chennai. Indian J Ophthalmol.

[7] Vijaya L (2008). Prevalence of primary angle-closure disease in an urban south Indian population and comparison with a rural population. The Chennai Glaucoma Study. Ophthalmology.

[8] Vijaya L (2008). Prevalence of primary open-angle glaucoma in an urban south Indian population and comparison with a rural population. The Chennai Glaucoma Study. Ophthalmology.

[9] Islam FM (2015). Factors Associated with Awareness, Attitudes and Practices Regarding Common Eye Diseases in the General Population in a Rural District in Bangladesh: The Bangladesh Population-based Diabetes and Eye Study (BPDES). PLoS One.

[10] Javadi, M.A., et al., Prevalence of diabetic retinopathy in Tehran province: a population-based study. BMC Ophthalmol, 2009. 9(1): p. 12-12. 10.1186/1471-2415-9-12 PMC2770536 19835608

[11] Fotouhi A, Hashemi H, Mohammad K (2006). Eye care utilization patterns in Tehran population: a population based cross-sectional study. BMC Ophthalmol.

[12] Katibeh M (2014). Knowledge and awareness of age related eye diseases: a population-based survey. J Ophthalmic Vis Res.

[13] Bakkar, M.M., et al., Epidemiology of symptoms of dry eye disease (DED) in Jordan: A cross-sectional non-clinical population-based study*.* Contact Lens and Anterior Eye, 2016(Journal Article). 10.1016/j.clae.2016.01.003 26833214

[14] Lau JT (2002). Knowledge about cataract, glaucoma, and age related macular degeneration in the Hong Kong Chinese population. Br J Ophthalmol.

[15] Sun J (2012). Prevalence and risk factors for primary open-angle glaucoma in a rural northeast China population: a population-based survey in Bin County, Harbin. Eye (Lond).

[16] Al-Bdour MD, Al-Till MI, Abu Samra KM (2008). Risk Factors for Diabetic Retinopathy among Jordanian Diabetics. Middle East Afr J Ophthalmol.

[17] Baker H, Murdoch IE (2008). Can a public health intervention improve awareness and health-seeking behaviour for glaucoma?. Br J Ophthalmol.

[18] Pfeiffer N, Krieglstein GK, Wellek S (2002). Knowledge about glaucoma in the unselected population: a German survey. J Glaucoma.

[19] Isawumi MA (2014). Awareness of and Attitude towards glaucoma among an adult rural population of Osun State, Southwest Nigeria. Middle East Afr J Ophthalmol.

[20] Uchino M (2013). Prevalence of dry eye disease and its risk factors in visual display terminal users: the Osaka study. Am J Ophthalmol.

[21] Rewri P, Kakkar M (2014). Awareness, knowledge, and practice: a survey of glaucoma in north Indian rural residents. Indian J Ophthalmol.

