# Supplementary material for: Public awareness of common eye diseases in Jordan
Source: BMC Ophthalmol. 2017 Oct 2;17:177. doi: 10.1186/s12886-017-0575-3 (PMC5625650; doi:10.1186/s12886-017-0575-3)
Supplement: Additional file 1: — Questionnaire. (DOCX 25 kb) [file 12886_2017_575_MOESM1_ESM.docx]

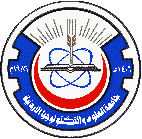


**Jordan University of Science and Technology**

1. **Name**
2. **Age** __________
3. **Gender** M \ F
4. **Level of education** 1. illetrate 2. Elementary level 3. high school (tawjihi)

4. University undergraduate (BSc) 5. Higher education (Master or PhD)

**5. Are you familiar with any eye disease? Can you describe the disease?**

Cataract________________________________________________________________________________

Glaucoma ______________________________________________________________________________

Diabetic retinopathy______________________________________________________________________

Dry eye________________________________________________________________________________

| **6. Can you report any risk factor associated with the disease?** | | | | | | | |
| --- | --- | --- | --- | --- | --- | --- | --- |
|  | Age | Smoking | Diet | Family history | Medication | Systemic condition  "HTN, DM" | other |
| - Cataract |  |  |  |  |  |  |  |
| - Glaucoma |  |  |  |  |  |  |  |
| - Diabetic retinopathy |  |  |  |  |  |  |  |
| - Dry eye |  |  |  |  |  |  |  |

**7. How do you know about the disease?**

Ophthalmology clinic

Optometry clinic

Family, friends, relatives

Media e.g. TV, radio

Reading e.g. book, newspaper, magazines

Internet

Previous history

Other _________________________________________________________________________________

| **8. About the condition** | | | | | | | | |
| --- | --- | --- | --- | --- | --- | --- | --- | --- |
|  | Is it a blinding condition | | Is it preventable | | Is it treatable | | Do you think the vision is back to normal after treatment | |
| - Cataract | **No (0)** | **Yes (1)** | **No (0)** | **Yes (1)** | **No (0)** | **Yes (1)** | **No (0)** | **Yes (1)** |
| - Glaucoma |  | |  | |  | |  | |
| - Diabetic retinopathy |  | |  | |  | |  | |
| - Dry eye |  | |  | |  | |  | |
